# Higher Mortality Associated With New-Onset Atrial Fibrillation in Cancer Patients: A Systematic Review and Meta-Analysis

**DOI:** 10.3389/fcvm.2022.867002

**Published:** 2022-04-14

**Authors:** Minha Murtaza, Mirza Mehmood Ali Baig, Jawad Ahmed, Liviu Ionut Serbanoiu, Stefan Sebastian Busnatu

**Affiliations:** ^1^Internal Medicine, Dow University of Health Sciences, Karachi, Pakistan; ^2^Department of Cardiology, Carol Davila University of Medicine and Pharmacy, Bucharest, Romania

**Keywords:** new-onset atrial fibrillation, cancer, mortality, cardio-oncology, meta-analysis

## Abstract

**Aim:**

This research was conducted to evaluate the mortality outcome of cancer patients with new-onset atrial fibrillation. We also aimed to assess if there was any confounding relation between the mortality of these patients and surgical intervention.

**Materials and Methods:**

A systemic search was conducted from electronic databases (PubMed/Medline, Cochrane Library, and Google Scholar) from inception to 7 February 2022. All statistical analyses were conducted in Review Manager 5.4.1. Studies meeting inclusion criteria were selected. Only those studies that involved cancer patients without pre-existing atrial fibrillation were selected, and mortality rate was compared between the patients who developed atrial fibrillation and those who did not. A random-effect model was used when heterogeneity was seen to pool the studies, and the result was reported in the odds ratio (OR) and the corresponding 95% confidence interval (CI).

**Results:**

Eighteen studies were selected for meta-analysis. Statistical analysis showed that the cancer patients who subsequently developed atrial fibrillation had a significantly higher mortality rate as compared to those who did not (OR = 1.90 [1.65, 2.19]; *p* < 0.00001; *I*^2^ = 100%). We also separately analyzed the mortality risk in the surgery group and the non-surgery group. Statistical analysis showed that there was significantly higher mortality rate associated with new-onset atrial fibrillation in cancer patients in the surgery group (OR= 3.68 [2.29, 5.94]; *p* < 0.00001; *I*^2^ = 61%) as well as in the non-surgery group (OR = 1.64 [1.39, 1.93]; *p* < 0.00001; *I*^2^ = 100%).

**Conclusion:**

Cancer patients, who subsequently developed atrial fibrillation, had a higher mortality rate as compared to those cancer patients who did not develop atrial fibrillation. A higher mortality rate was seen in both surgical and non-surgical subgroups. This implies that extra care and specific measures must be taken in the management of cancer patients with new-onset atrial fibrillation.

## Introduction

Cancer is among the most terrible diseases in the world, and its incidence is constantly on the rise (1). Considering the prevalence of known risk factors such as aging, family history, obesity, and radiation exposure, and increased adoption of unhealthy lifestyles such as tobacco smoking, alcohol, physical inactivity, and unhealthy diet, the rate of occurrence of almost all types of cancer is expected to further increase (2).

Lung cancer and breast cancer were found to be the most frequently diagnosed cancers and the leading cause of death among males and females, respectively, in 2012. Esophageal cancer notably varies in incidence rates internationally, and is found to be higher in men. Colorectal cancer is the third most frequently diagnosed cancer in males, and the second-most in females. Non-Hodgkin lymphoma is more common in more developed areas, with the highest incidence rates found in Australia, Western and Northern Europe, and Northern America (2).

Atrial fibrillation is the most frequently encountered cardiac arrhythmia that has great clinical importance. It causes significant morbidity and mortality by increasing the incidence of cardiomyopathies and their subsequent complications (3). It is well established that cancer patients undergoing surgery have an increased risk of developing atrial fibrillation in the perioperative and postoperative periods (4, 5).

In a meta-analysis conducted by Yuan et al., it was demonstrated that there is a higher risk of developing atrial fibrillation in cancer patients as compared to non-cancer patients. Cancer patients have a 47% higher risk of atrial fibrillation (6). The risk of atrial fibrillation varies in different types of cancer. For instance, colorectal cancer patients have a 54% higher risk and breast cancer patients have double the risk of developing atrial fibrillation as compared to people with no cancer (6).

Conversely, an increased risk of subsequent diagnosis of cancer in patients with atrial fibrillation has also been reported (7).

It has been found that cancer-related atrial fibrillation occurs more often after cancer surgery. Many risk factors have been implicated in the development of postoperative atrial fibrillation. These include advanced age, male gender, and advanced cancer stage (8). Moreover, a study reported 19.6% incidence rate of postoperative atrial fibrillation in patients undergoing an operation for malignant pulmonary disease, compared to the 3.1% incidence rate in those operated for benign pulmonary disease. This suggests that atrial fibrillation is not merely a complication of surgery, but it has a strong link with cancer itself (9).

In the case of surgical patients, pre-operative cardiac symptoms and echocardiogram (ECG) abnormalities, operative parameters, and post-operative clinical findings may be responsible for the development of atrial fibrillation, but their causal role could not be demonstrated (9).

Cancer could not be demonstrated as an independent predictor of atrial fibrillation. However, the elevated levels of C-reactive protein (CRP) associated with cancer, and the remodeling of the atrial structure due to the presence of an inflammatory state suggest inflammation as the causal intermediary link between the two (10).

Increased incidence of atrial fibrillation in cancer patients may also be related to autonomic disturbances, atrial inflammation due to autoimmune paraneoplastic syndromes, or cancer therapy (10, 11).

The previous meta-analysis conducted by Yuan et al. determined the relationship between cancer and the risk of developing atrial fibrillation, but it did not evaluate the impact of atrial fibrillation on cancer mortality (6). There has been inconsistent evidence on whether the new-onset atrial fibrillation in cancer patients significantly affects mortality outcomes. Therefore, this systematic review and meta-analysis was conducted to establish a conclusive relationship between new-onset atrial fibrillation and mortality in cancer patients.

## Materials and Methods

### Data Sources and Search Strategy

This systematic review and meta-analysis was conducted according to the Preferred Reporting Items for Systematic Review and Meta-Analyses (PRISMA) guidelines (12). An electronic search from PubMed/Medline, Cochrane Library, and Google Scholar was conducted from their inception to 7 February 2022 (detailed strategy is provided in Table 1), with only English language-based literature, using the search string: (cancer^*^ OR carcinoma^*^ malignancy^*^) AND (atrial fibrillation OR AF OR a-fibrillation) AND (mortality OR death OR survival). In addition, we manually screened the cited articles of previous meta-analyses, cohort studies, and review articles to identify any relevant studies.

**Table 1:** Search strategy.

**Search engine**	**Search strategy**
Pubmed/medline	(“cancer*”[All Fields] OR (“carcinoma*”[All Fields] AND “malignanc*”[All Fields])) AND (“atrial fibrillation”[MeSH Terms] OR (“atrial”[All Fields] AND “fibrillation”[All Fields]) OR “atrial fibrillation”[All Fields] OR “AF”[All Fields] OR “a-fibrillation”[All Fields]) AND (“mortality”[MeSH Terms] OR “mortality”[All Fields] OR “mortalities”[All Fields] OR “mortality”[MeSH Subheading] OR (“death”[MeSH Terms] OR “death”[All Fields] OR “deaths”[All Fields]) OR (“mortality”[MeSH Subheading] OR “mortality”[All Fields] OR “survival”[All Fields] OR “survival”[MeSH Terms] OR “survivability”[All Fields] OR “survivable”[All Fields] OR “survivals”[All Fields] OR “survive”[All Fields] OR “survived”[All Fields] OR “survives”[All Fields] OR “surviving”[All Fields]))
Cochrane	(cancer* OR carcinoma* malignanc*) AND (atrial fibrillation OR AF OR a-fibrillation) AND (mortality OR death OR survival)
Google scholar	(cancer* OR carcinoma* malignanc*) AND (atrial fibrillation OR AF OR a-fibrillation) AND (mortality OR death OR survival)

### Study Selection

All studies were included, which met the following eligibility described as PECOS: (1) P (Population): cancer patients; (2) E (Exposure): atrial fibrillation; (3) C (Control): cancer patients without atrial fibrillation; (4) O (Outcome): mortality; (5) S (Studies): human-based randomized controlled trials and cohort studies published in English only.

Case series, case reports, literature reviews, editorials, and studies not meeting the inclusion criteria were excluded.

### Data Extraction and Quality Assessment of Studies

Two reviewers independently searched electronic databases. Studies searched were exported to the EndNote Reference Library software version 20.0.1 **(**Clarivate Analytics), and duplicates were screened and removed.

Data extraction and quality assessment of included studies were done simultaneously and independently by two reviewers. Newcastle-Ottawa Scale (NOS) was used to assess the quality of the cohort studies. NOS score <6 was considered high risk for bias, 6–7 was moderate, and score >7 was considered low risk of bias (details of scoring is provided in Table 2). The modified Cochrane Collaboration's risk of bias tool for randomized controlled trials was used to assess the quality of published trials (details are provided in Table 3).

**Table 2:** Quality assessment of cohorts using New Ottawa scale (NOS).

**Studies**	**Selection (maximum 4)**				**Comparability (maximum 2)**	**Outcome (maximum 3)**			**Total score**
	**Representativeness of the exposed cohort**	**Selection of the non-exposed cohort**	**Ascertainment of exposure**	**Demonstration that outcome of interest was not present at start of study**	**Comparability of cohorts on the basis of the design or analysis**	**Assessment of outcome**	**Was follow-up long enough for outcomes to occur**	**Adequacy of follow up of cohorts**	
Amar et al. (14)	1	0	1	1	1	1	1	1	7
Amioka et al. (15)	1	1	1	1	1	1	1	1	8
Cardinale et al. (16)	1	1	1	1	1	1	1	1	8
Chin et al. (17)	1	1	1	1	2	1	1	1	9
Constantin et al. (18)	1	1	1	1	2	1	1	1	9
Imperatori et al. (19)	1	1	1	1	1	1	1	1	8
Ishibashi et al. (20)	1	1	1	1	2	1	1	1	9
Kotova et al. (21)	1	1	1	1	1	1	1	1	8
McComrack et al. (22)	1	1	1	1	2	1	1	1	9
Murthy et al. (23)	1	1	1	1	1	1	1	1	8
Rao et al. (25)	1	1	1	1	2	1	1	1	9
Roselli et al. (26)	1	0	1	1	1	1	1	1	7
Stawicki et al. (27)	1	1	1	1	2	1	1	1	9
Wang et al. (28)	1	1	1	1	2	1	1	1	9
Ammad Ud Din et al. (29)	1	1	1	0	2	1	1	1	8
Han et al. (30)	1	1	1	1	2	1	1	1	9
Zubair Khan et al. (31)	1	1	1	1	2	1	1	1	9

**Table 3:** Quality assessment of clinical trials using Cochrane Collaboration's risk of bias tool.

**Study**	**Adequate sequence generation**	**Allocation concealment**	**Blinding of participants and personnel**	**Blinding of outcome assessment**	**Incomplete outcome data**	**Selective outcome reporting**	**Free of other bias**	**Net risk**
Ojima et al. (24)	High risk	Unclear risk	High risk	Unclear risk	Low risk	High risk	High risk	High risk

### Statistical Analysis

Review Manager (version 5.4.1; Copenhagen: The Nordic Cochrane Center, The Cochrane Collaboration, 2020) was used for all statistical analyses. The data from studies were pooled using a random-effects model. Analysis of results was done by calculating the odds ratio (OR) with respective 95% confidence intervals (CI). The chi-square test was performed to assess any differences between the subgroups. Sensitivity analysis was done to see if any individual study was driving the results and to implore reasons for high heterogeneity. As per Higgins et al., scale for heterogeneity was considered as follows: I^2^ = 25–60%–moderate; 50–90%–substantial; 75–100%–considerable heterogeneity, and *p* < 0.1 indicated significant heterogeneity (13). A value of *p* < 0.05 was considered significant for all analyses.

## Results

### Literature Search Results

**Figure 1:**
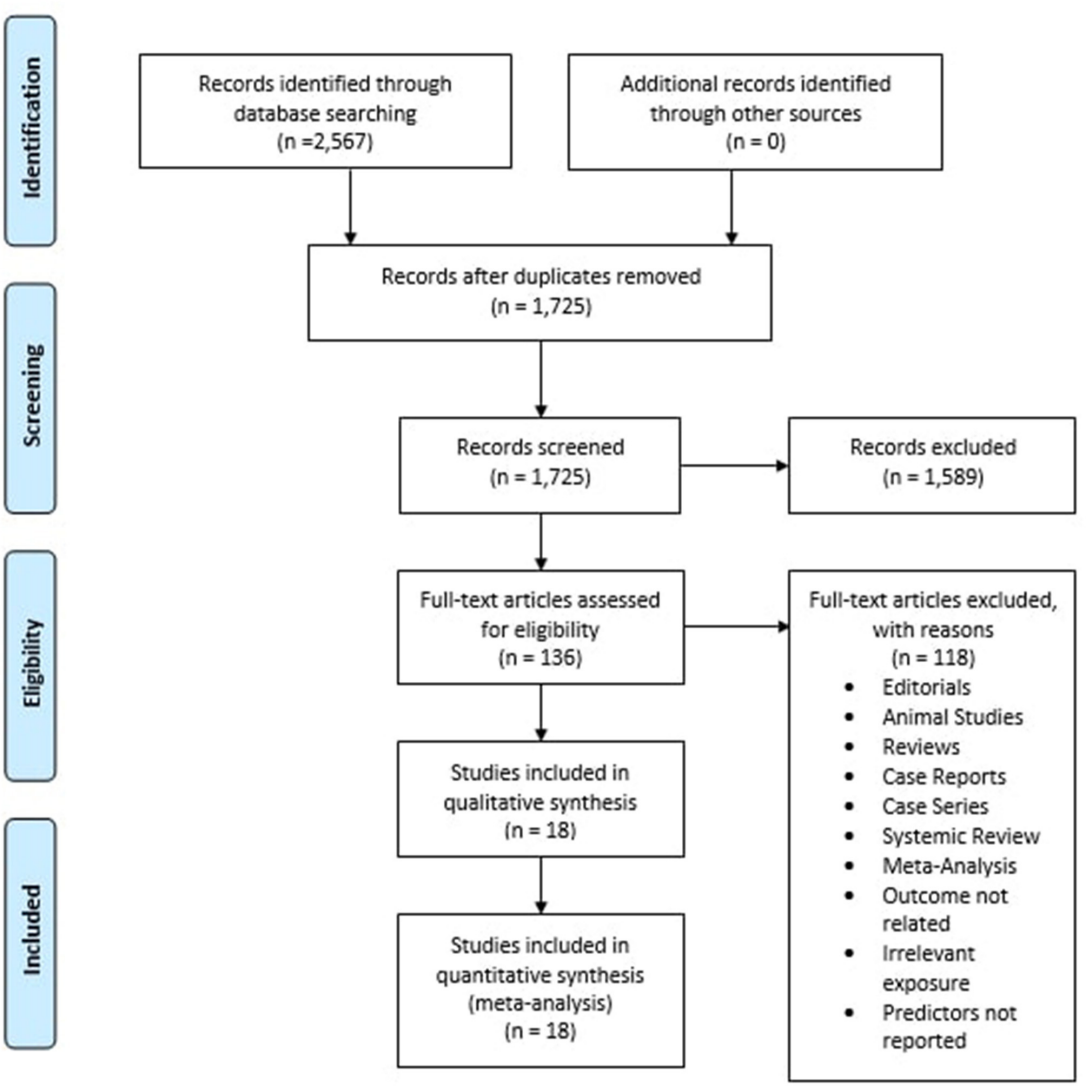
Prisma flow chart summarizing the literature search.

The initial search of the three electronic databases yielded 2,567 potential studies. After exclusions based on titles and abstracts, the full texts of 136 studies were read for possible inclusion. A total of 18 studies remained for quantitative analysis. Figure 1 summarizes the results of our literature search.

### Study Characteristics

Table 4 provides the basic characteristics of included studies (14–31). Our analysis included 18 published studies. Among these, there are 17 observational studies and 1 randomized controlled trial. The studies were conducted in different regions of the world, i.e., USA, Japan, Italy, Korea, Romania, Ireland, China, and the UK. A total of 6 studies evaluated lung cancer, 5 studies evaluated esophageal cancer, and 6 studies evaluated various other cancers including lymphoma, leukemia, colorectal cancer, prostate cancer, and breast cancer. The mean age of patients was 67.12 years.

**Table 4:** Characteristics of included studies.

**Study**	**Year**	**Study design**	**Duration**	**Country**	**Total cancer patients (n)**	**Males (n)**	**Mean age (years)**	**Incident AF (n)**	**Cancer type**	**Mortality attestation**	**Major causes of death**	**Surgery or non-surgery group**	**Risk of Bias**
Amar et al. (14)	2002	Cohort	1990–1999	USA	527	325	68 (AF) 62 (non-AF)	79	Lung cancer, Esophageal cancer	Medical record	Surgery-related complications	Surgery	Moderate Risk
Amioka et al. (15)	2016	Cohort	2009–2013	Japan	249	121	67	15	Hodgkin, Non-Hodgkin lymphoma	Medical record	Primary disease, solid cancer, hepatitis, sepsis	Non-surgery	Low Risk
Cardinale et al. (16)	1999	Cohort	1995–1997	Italy	233	170	59.3	28	Lung cancer	Medical record	Surgery-related complications	Surgery	Low Risk
Chin et al. (17)	2016	Cohort	2005–2012	Korea	583	548	67 (AF) 62 (non-AF)	63	Esophageal cancer	Medical record	Surgery-related complications	Surgery	Low Risk
Constantin al (18)	2020	Cohort	2008–2017	Romania	391	N/A*	N/A*	27	Colorectal cancer	Medical record	Surgery-related complications	Surgery	Low Risk
Imperatori et al. (19)	2012	Cohort	1996–2009	Italy	454	369	65.4	45	Lung cancer	Medical record	Surgery-related complications	Surgery	Low Risk
Ishibashi et al. (20)	2020	Cohort	2010–2019	Japan	947	626	69.2	49	Lung cancer	Medical record	Surgery-related complications	Surgery	Low Risk
Kotova et al. (21)	2017	Cohort	2005–2014	USA	933	426	72 (AF) 65.4 (non-AF)	113	Lung cancer	Medical record	Surgery-related complications	Surgery	Low Risk
McComrack et al. (22)	2014	Cohort	2006–2013	Ireland	473	344	63	96	Esophageal cancer, Junctional cancer	Medical record	Surgery-related complications	Surgery	Low Risk
Murthy et al. (23)	2003	Cohort	1982–2000	China	288	235	66.8 (AF) 67 (non-AF)	144	Esophageal cancer	Medical record	Surgery-related complications	Surgery	Low Risk
Ojima et al. (24)	2020	RCT*	2014–2016	Japan	57	77	N/A*	13	Esophageal cancer	Medical record	Surgery-related complications	Surgery	High Risk
Rao et al. (25)	2012	Cohort	1991–2009	UK	997	709	67	209	Esophageal cancer	Medical record	Surgery-related complications	Surgery	Low Risk
Roselli et al. (26)	2005	Cohort	1998–2002	USA	183	N/A*	N/A*	91	Lung cancer	Medical record	Surgery-related complications	Surgery	Moderate Risk
Stawicki et al. (27)	2011	Cohort	1996–2007	USA	156	145	63.7 (AF) 59.8 (non-AF)	32	Esophageal cancer	Medical record	Surgery-related complications	Surgery	Low Risk
Wang et al. (28)	2021	Cohort	2013–2018	China	324	291	58.4	75	Lung cancer	Medical record	Surgery-related complications	Surgery	Low Risk
Ammad Ud Din et al. (29)	2021	Cohort	2009–2018	USA	14,530	8,569	81.80 (AF) 81.76 (non-AF)	7,265	chronic lymphocytic leukemia	Medical record	Acute Myocardial Infarction, heart failure, cardiac arrest, stroke	Non-surgery	Low Risk
Han et al. (30)	2021	Cohort	2003–2014	China	2,478,598	1,104,903	N/A	216,737	Various cancers	Medical record	Not specified	Non-surgery	Low Risk
Zubair Khan et al. (31)	2021	Cohort	2005–2015	USA	46,030,380	7,673,063	N/A	6,731,310	Various cancers	Medical record	Not specified	Non-surgery	Low Risk

N/A*, Not available; RCT*, Randomized Controlled Trial.

### Publication Bias and Quality Assessment

**Figure 2:**
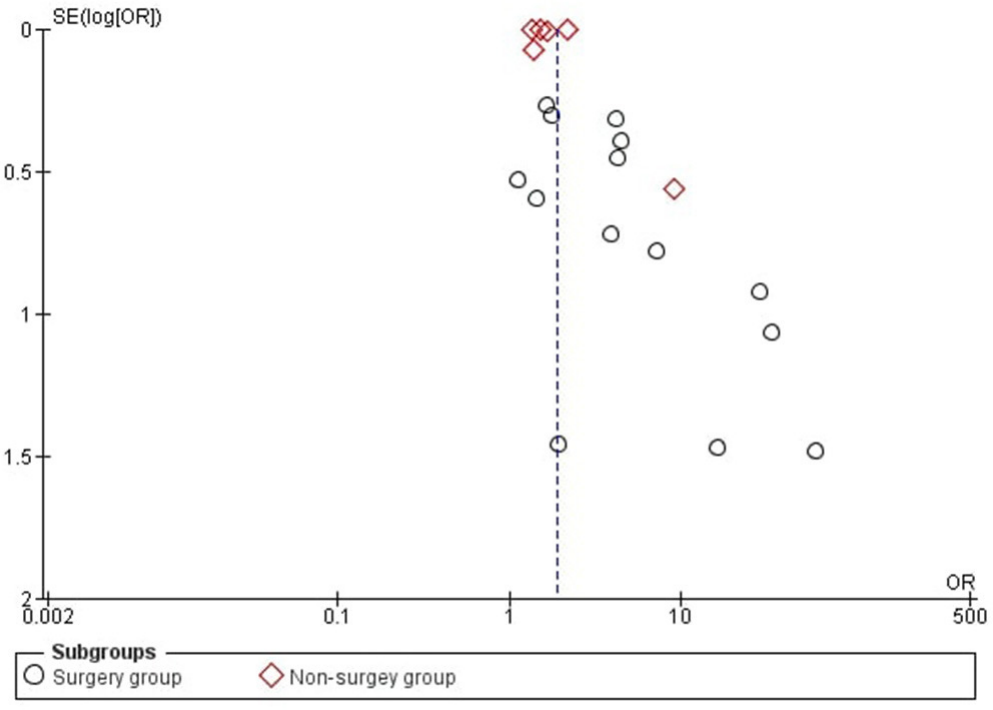
Funnel plot which is used to demonstrate publicaion bias.

The visual inspection of the funnel plot (Figure 2) did indicate that there is publication bias in our meta-analysis.

Out of the 18 studies, 15 studies have a low risk of bias (15–23, 25, 27–31), two studies have a moderate risk of bias (14, 26), and one study has a high risk of bias (24).

### Results of Meta-Analysis

**Figure 3:**
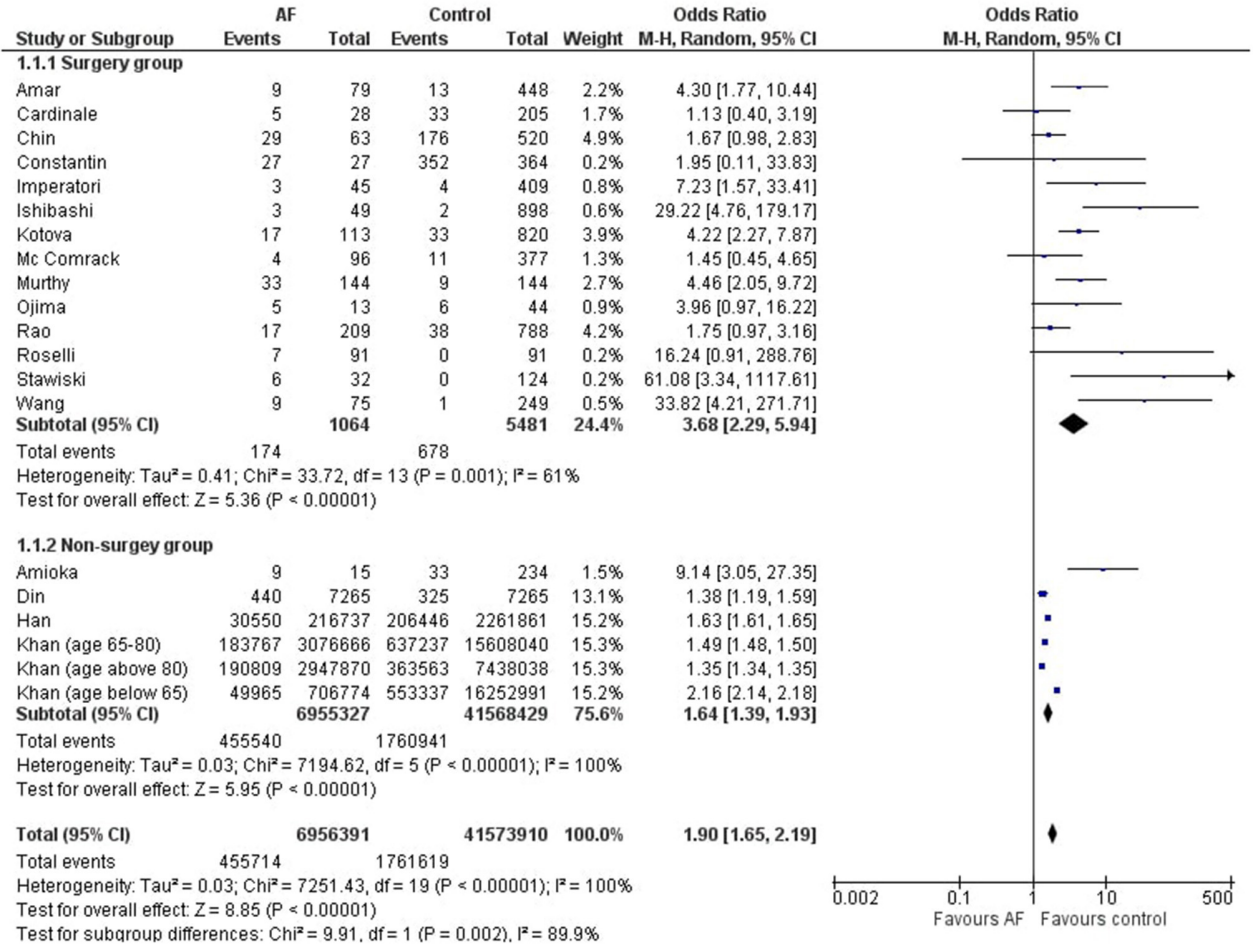
Forest plot showing effect size of mortality outcome of atrial fibrillation in different types of cancer and also its overall affect.

Detailed forest plots, outlining the effect size of overall mortality outcome of atrial fibrillation in cancers, are shown in Figure 3. A forest plot outlining the effect size based on surgery and the non-surgery group is also presented in Figure 3.

#### Overall Mortality Outcome of Atrial Fibrillation in Cancers

Eighteen studies evaluated the mortality outcome for cancer patients who develop atrial fibrillation. Table 5 provides further details of the studies selected for this objective. Pooled results (Figure 3) showed a significantly higher mortality rate in cancer patients who subsequently develop atrial fibrillation as compared to those in the control group (OR = 1.90 [1.65, 2.19]; *p* < 0.00001; *I*^2^ = 100%).

**Table 5:** Analytical details of the studies that were selected.

**Study name and year**		**Deaths in AF**		**Deaths in control**		**Odds ratio [95% CI]**	***P* value**
		**Events (n)**	**Total (n)**	**Events (n)**	**Total (n)**		
Amar et al. (14)	2002	9	79	13	448	4.30 [1.77, 10.44]	0.0013
Amioka et al. (15)	2016	9	15	33	234	9.14 [3.05, 27.35]	0.0001
Cardinale et al. (16)	1999	5	28	33	205	1.13 [0.40, 3.19]	0.8132
Chin et al. (17)	2016	29	63	176	520	1.67 [0.98, 2.83]	0.0576
Constantin et al. (18)	2020	27	27	352	364	1.95 [0.11, 33.83]	0.6463
Imperatori et al. (19)	2012	3	45	4	409	7.23 [1.57, 33.41]	0.0113
Ishibashi et al. (20)	2020	3	49	2	898	29.22 [4.76, 179.17]	0.0003
Kotova et al. (21)	2017	17	113	33	820	4.22 [2.27, 7.87]	0.0000
McComrack et al. (22)	2014	4	96	11	377	1.45 [0.45, 4.65]	0.5352
Murthy et al. (23)	2003	33	144	9	144	4.46 [2.05, 9.72]	0.0002
Ojima et al. (24)	2020	5	13	6	44	3.96 [0.97, 16.22]	0.0559
Rao et al. (25)	2012	17	209	38	788	1.75 [0.97, 3.16]	0.0652
Roselli et al. (26)	2005	7	91	0	91	16.24 [0.91, 288.76]	0.0576
Stawicki et al. (27)	2011	6	32	0	124	61.08 [3.34, 1,117.61]	0.0056
Wang et al. (28)	2021	9	75	1	249	33.82 [4.21, 271.71]	0.0009
Ammad Ud Din et al. (29)	2021	440	7,265	325	7,265	1.38 [1.19, 1.59]	0.0000
Han et al. (30)	2021	30,550	2,16,737	2,06,446	2,261,861	1.63 [1.61, 1.65]	0.0000
Zubair Khan et al. (31) (age 65-80)	2021	1,83,767	30,76,666	6,37,237	15,608,040	1.49 [1.48, 1.50]	0.0000
Zubair Khan (31) (age below 65)	2021	1,90,809	29,47,870	3,63,563	74,38,038	1.35 [1.34, 1.35]	0.0000
Zubair Khan (31) (age above 80)	2021	49,965	7,06,774	5,53,337	1,62,52,991	2.16 [2.14, 2.18]	0.0000

#### Surgery Group

Out of 18 studies, 14 studies reported data of patients undergoing cancer-related surgeries. Statistical analysis showed that there was a significantly higher mortality rate in cancer patients who subsequently develop atrial fibrillation as compared to those in the control group (OR = 3.68 [2.29, 5.94]; *p* < 0.00001; *I*^2^ = 61%).

#### Non-Surgery Group

Out of 18 studies, 4 studies reported data of non-surgical patients having cancer. Statistical analysis showed that there was a significantly higher mortality rate in cancer patients who subsequently develop atrial fibrillation as compared to those in the control group (OR = 1.64 [1.39, 1.93]; *p* < 0.00001; *I*^2^ = 100%).

### Sensitivity Analysis

A sensitivity analysis was conducted to assess the influence of each study on the overall effect, by excluding one study at a time, followed by the generation of pooled OR for the rest of the studies. No significant change was observed after the exclusion of any individual study, suggesting that the results were robust.

We also removed the study by Amioka et al. (15) to check its effect on our results, as it was the only study with a high risk of bias, but there was no statistically significant change. The overall result was OR = 1.85 [1.61, 2.14]; *p* < 0.0001; *I*^2^= 100%.

## Discussion

Our systematic review and meta-analysis of 18 published studies suggest that cancer patients who subsequently develop atrial fibrillation have an increased rate of mortality as compared to those who do not (OR = 1.90 [1.65, 2.19]; *p* < 0.00001; *I*^2^ = 100%). We also separately analyzed the mortality risk in the surgery group and the non-surgery group. This subgroup analysis shows that new-onset atrial fibrillation associated with cancer increases the mortality rate, irrespective of any surgical intervention. Mechanisms contributing to death included surgery-related complications, sepsis, hepatitis, pneumonia, heart failure, myocardial infarction, and stroke.

Previous studies have evaluated this relationship in specific cancer patients. However, the results are inconsistent, and the studies are limited by their sample size and geographical location. We have pooled data from studies performed in different regions of the world and with different cancer types to provide better and more reliable evidence.

Cancer continues to be one of the deadliest diseases worldwide. In the year 2020, an estimated 19.3 million new cancer cases and about 10 million cancer deaths occurred globally (32). Death in cancer patients may occur due to index cancer, non-index cancer, or non-cancer causes. Patients with cancers of the colorectum, prostate, breast, genitourinary tract, tonsils, melanoma, and lymphomas are more likely to die due to non-cancer causes. Heart disease is the most common non-cancer cause of death (33). Many mechanisms can lead to the death of cancer patients, including infections (36%), hemorrhagic and thromboembolic phenomena (18%), and respiratory failure (19%) (34).

It is well recognized that there is an increased risk of developing atrial fibrillation in cancer patients (6). Cancer itself is a high-risk and life-threatening condition. Concomitant development of atrial fibrillation brings additional risks including infections, stroke, and bleeding among others (35). Atrial fibrillation alone is associated with four times increased risk of all-cause mortality as compared to the general population (36). Atrial fibrillation has been demonstrated as a risk factor for stroke (37). A five-fold increased risk of stroke has been reported to be associated with chronic atrial fibrillation (38).

The underlying mechanism leading to the development of atrial fibrillation in cancer patients has been a matter of concern. Atrial fibrillation may be a co-morbid state considering the common predisposing factors for both conditions, and it might involve specific etiologies (10).

Inflammation of the atria due to autoimmune paraneoplastic syndromes, the abnormal release of some hormones by cancer cells, and imbalances between the sympathetic and parasympathetic autonomic control may predispose the patient to atrial fibrillation (10). These mechanisms support our findings that new-onset atrial fibrillation can increase the risk of mortality in cancer patients.

Cancer therapy may be responsible for the development of atrial fibrillation. However, it has been observed that the risk of developing atrial fibrillation persists even if no cancer-specific treatment has been given (11).

Although the exact mechanism responsible for increased mortality in cancer patients with atrial fibrillation remains undetermined, it can be speculated from the following findings. In atrial fibrillation, the perfusion of tissues becomes inadequate, and due to this, oxidative injury can occur (38, 39). The hypoxic conditions not only favor tumor survival and growth but also cause resistance to radiation therapy (40, 41). Moreover, endothelial dysfunction, inflammatory conditions, and prothrombic state associated with atrial fibrillation may also be responsible for the worsened outcomes (42). The inflammatory state may be further aggravated by cancer surgery. The increase in in-hospital mortality can also be explained by the fact that patients with atrial fibrillation are susceptible to hospital-acquired pneumonia, which can become the cause of death in these patients (43).

Most of the studies included in our analysis enrolled patients undergoing cancer surgery. It is highly likely that the surgery may play an important role in the worsened outcomes. Various processes and mediators involved in surgical wound healing may accelerate tumor growth, invasion, and metastasis (44). Tumor outgrowth may be caused by activation of epithelial, endothelial, and inflammatory cells; platelets, and fibroblasts; and production of growth factors and cytokines during the healing of surgical wounds (45).

The efficacy of different prophylactic approaches to prevent atrial fibrillation after lung surgery was evaluated in a meta-analysis by Zhang et al. It was found that amiodarone was the most effective in preventing postoperative atrial fibrillation (46). The use of oral anticoagulants is associated with a lower risk of mortality in patients with atrial fibrillation (36). It has been demonstrated that the patients who have concomitant cancer and atrial fibrillation can be benefited from anticoagulation with nonvitamin K antagonist oral anticoagulants (also known as direct oral anticoagulants (DOACs)) as well as warfarin (47). However, DOACs have a safer profile and greater effectiveness as compared to vitamin K antagonists (VKAs) (48). Moreover, CHADS2 and CHA2DS2-VASc scores can be used as tools to predict the risk of stroke and mortality in patients with cancer and atrial fibrillation and guide in decision-making accordingly (49).

Although it is suggested that atrial fibrillation after cancer surgery tends to be transient with few clinical outcomes and does not require prolonged monitoring and intensive care (5), recent data suggest a relationship between atrial fibrillation and increased mortality in cancer patients. Cancer patients who subsequently develop atrial fibrillation may need closer surveillance during follow-up, and specific strategies to manage atrial fibrillation should be included in the cancer management plan.

### Limitation

Our study is limited in several ways: (a) The majority of the studies were observational, the results of which can have some bias. (b) Most of the studies enrolled patients undergoing surgery, so our outcome, i.e., increased mortality, may not solely be due to the atrial fibrillation but there might be some role of the after-effects of surgery. (c) High heterogeneity was seen in our results because we pooled different studies containing different cancer types. These studies were pivotal in forming analysis, but more studies with the community and random controls should be conducted.

## Conclusion

Cancer patients who subsequently developed atrial fibrillation had a higher mortality rate as compared to those cancer patients who did not develop atrial fibrillation. A higher mortality rate was seen in both surgical and non-surgical subgroups. This implies that cancer patients who subsequently develop atrial fibrillation need to be followed up with careful monitoring of the arrhythmia, and specific measures should be taken to minimize the adverse outcomes brought on by it.

## Data Availability Statement

The original contributions presented in the study are included in the article/supplementary material, further inquiries can be directed to the corresponding author/s.

## Author Contributions

MB worked alongside MM in all steps from literature search till manuscript preparation. JA reviewed and actively participated in the analysis, manuscript preparation and reviewing, and is designated as the senior author. LS and SB played role in final analysis and reviewing of the manuscript. All authors contributed to the article and approved the submitted version.

## Funding

The Open Access publication fee has been funded by Liviu Ionut Serbanoiu (LS).

## Conflict of Interest

The authors declare that the research was conducted in the absence of any commercial or financial relationships that could be construed as a potential conflict of interest.

## Publisher's Note

All claims expressed in this article are solely those of the authors and do not necessarily represent those of their affiliated organizations, or those of the publisher, the editors and the reviewers. Any product that may be evaluated in this article, or claim that may be made by its manufacturer, is not guaranteed or endorsed by the publisher.
